# Quantitative Evaluation of the Inhibitory Effects of Commercially Available Probiotics on Dual-Species Biofilms in Root Canals: A qPCR-Based Short-Term In Vitro Study

**DOI:** 10.3390/antibiotics15040354

**Published:** 2026-03-30

**Authors:** Beyza Yalçıntaş, Yakup Üstün, Nurbanu Yaşar, Seda Tezcan Ülger, Gönül Aslan, Bertan Kesim

**Affiliations:** 1 Department of Endodontics, Faculty of Dentistry, Erciyes University, Kayseri 38039, Turkey; 2 Department of Medical Microbiology, Faculty of Medicine, Mersin University, Mersin 33343, Turkey; 3 Department of Endodontics, Faculty of Dentistry, Nuh Naci Yazgan University, Kayseri 38170, Turkey

**Keywords:** probiotics, root canal, biofilm, qPCR

## Abstract

**Objectives:** To quantitatively evaluate the inhibitory effects of commercially available probiotic formulations (Probien, Enterogermina, Reflor) applied as intracanal medicaments against mature dual-species biofilms of *Enterococcus faecalis* (*E. faecalis*) and *Candida albicans* (*C. albicans*) using a qPCR-based in vitro root canal model, with calcium hydroxide included as the reference intracanal medicament for comparison. **Materials and Methods:** Root canal specimens containing mature dual-species biofilms were medicated with probiotic–poloxamer gel formulations (Probien, Enterogermina, or Reflor) or calcium hydroxide (reference inhibitory control); infected but untreated canals served as the non-inhibitory control, and sterile non-inoculated specimens were included to confirm procedural sterility. After a 7-day intracanal application period, microbial loads were quantified at baseline and post-treatment by qPCR, and results were expressed as delta cycle threshold (ΔCt), colony-forming equivalents (CFE/mL), and percentage reduction values. **Results:** A total of 78 specimens (n = 13 per group) were analyzed. No significant intergroup differences were found in *E. faecalis* ΔCt or reduction percentages (*p* > 0.05), indicating its persistence despite intracanal medication. For *C. albicans*, differences among groups were significant (*p* < 0.001). Calcium hydroxide showed the strongest antifungal effect, producing marked ΔCt and CFE reductions versus probiotic and positive control groups, whereas probiotic formulations displayed only limited antifungal activity and no measurable inhibition against *E. faecalis*. **Conclusions:** Under the conditions of this in vitro model, the tested commercially available probiotic formulations—originally developed for gastrointestinal use—did not demonstrate significant antimicrobial effects against mature *E. faecalis*–*C. albicans* biofilms. These findings should be interpreted in the context of the absence of probiotic formulations specifically designed for intracanal use and the distinct ecological characteristics of the root canal system, which represents a closed, low-oxygen environment dominated by hard-tissue surfaces. Rather than excluding the potential of probiotics in endodontics, the present results highlight the need for root canal–adapted probiotic strains and delivery strategies tailored to intracanal conditions. **Clinical Relevance:** This in vitro study provides experimental insight into the limitations of directly applying commercially available gastrointestinal probiotic formulations within the root canal system. The findings highlight the importance of developing root canal–specific probiotic strains and delivery strategies tailored to the unique ecological conditions of the intracanal environment, thereby informing future translational and experimental research in biological endodontics.

## 1. Introduction

Persistent endodontic infections continue to pose a significant clinical challenge, contributing to treatment failures and necessitating retreatment in a substantial number of cases [1]. Microorganisms find refuge in remote areas of the root canal system, including isthmuses, lateral canals, and dentinal tubules, causing persistent infections to be remarkably hard to eliminate, primarily because the complex internal architecture of these sites restricts mechanical instrumentation [2], reduces irrigant flow [3], and facilitates biofilm formation that protects microbes from host immune defenses [4]. Persistent endodontic infections are frequently associated with *Enterococcus faecalis* (*E. faecalis*) [5] and *Candida albicans* (*C. albicans*) [6], whose biofilm-associated phenotype exhibits pronounced resistance to both routine chemomechanical debridement and intracanal therapies. Their ability to survive harsh environmental conditions [7,8], form robust biofilms [4,9], and invade dentinal tubules [10,11] underscores the need for novel antimicrobial strategies that can penetrate these complex niches and disrupt multispecies biofilms more effectively. In clinical cases where microbial load is expected to be high—such as in teeth with necrotic pulps or periapical lesions [12]—and where complex anatomical irregularities hinder complete disinfection, intracanal medicaments are generally considered beneficial to complement conventional chemomechanical preparation and improve treatment outcomes [13].

Calcium hydroxide is widely used in endodontics as a long-term intracanal medicament owing to its high alkalinity and broad-spectrum antimicrobial activity and is commonly applied in powder–liquid or premixed paste formulations. In the present study, it was selected as the sole comparator as it represents the most clinically established reference intracanal medicament for long-term disinfection, enabling biologically relevant comparison with experimental probiotic-based formulations under standardized in vitro conditions. Despite its widespread use, calcium hydroxide is associated with several well-documented limitations in long-term antimicrobial effectiveness. These limitations are largely attributed to the buffering effect of dentin, which can neutralize the hydroxyl ions responsible for its antimicrobial action [11], thereby reducing its efficacy against resistant endodontic pathogens. Additionally, prolonged placement of calcium hydroxide within the root canal system has been associated with alterations in dentin structure and increased risk of root fractures, raising concerns about its safety in extended treatments [14]. Moreover, calcium hydroxide may not exert sufficient antimicrobial activity against all endodontic pathogens [15,16] and has shown variable sealing ability within the root canal system, potentially contributing to reinfection [17].

In light of these shortcomings, alternative biologically based strategies such as probiotics have gained increasing attention. Probiotics, defined as live microorganisms that confer health benefits to the host, exhibit promising antibacterial activity against oral pathogens by competing for nutrients, producing inhibitory substances, and modulating host immune responses [18]. Recent evidence suggests that probiotic strains, particularly from the *Lactobacillus* and *Bifidobacterium* genera, exhibit notable antimicrobial effects against *E. faecalis*, reflected by reductions in microbial load, while demonstrating moderate inhibitory effects against *C. albicans*, supporting their potential as adjuncts or alternatives to conventional intracanal medicaments [19,20,21].

Despite encouraging preliminary findings, to the best of our knowledge, no study has quantitatively assessed the antimicrobial efficacy of intracanal probiotic applications on dual-species biofilms of *E. faecalis* and *C. albicans* using molecular microbiological techniques.

At the time of study design, no probiotic formulations specifically developed for intracanal or oral endodontic applications were commercially available. Therefore, commercially available gastrointestinal probiotic formulations were selected as a standardized and pragmatic model for exploratory investigation. These products were chosen based on their clinical availability, established safety profiles, and well-defined microbial composition—including *Lactobacillus*, *Bifidobacterium*, *Bacillus*, and *Saccharomyces* strains previously investigated for antimicrobial activity against oral pathogens. These formulations were used as a proof-of-concept model to evaluate their intrinsic antimicrobial potential within the unique ecological conditions of the root canal system, while also identifying limitations that may guide the development of root canal–specific probiotic strategies.

Accordingly, this in vitro study aimed to investigate the inhibitory effects of selected commercial probiotic formulations (Enterogermina, Reflor, Probien) against dual-species biofilms formed within root canals, utilizing a qPCR-based quantification method. This molecular approach provides enhanced sensitivity and specificity for microbial detection compared to traditional culture-based techniques.

The null hypothesis of this in vitro study posits that commercial probiotic formulations have no significant inhibitory effect on *E. faecalis*–*C. albicans* dual-species biofilms. By testing this hypothesis, the present study aims to provide foundational evidence regarding the antimicrobial behavior of currently available probiotic formulations within an intracanal environment. Rather than proposing these formulations as direct alternatives to established intracanal medicaments such as calcium hydroxide, this approach seeks to explore probiotic-based strategies for modulating root canal microbiota and to inform the rational development of root canal–adapted probiotic formulations for persistent endodontic infections.

## 2. Materials and Methods

**Figure 1:**
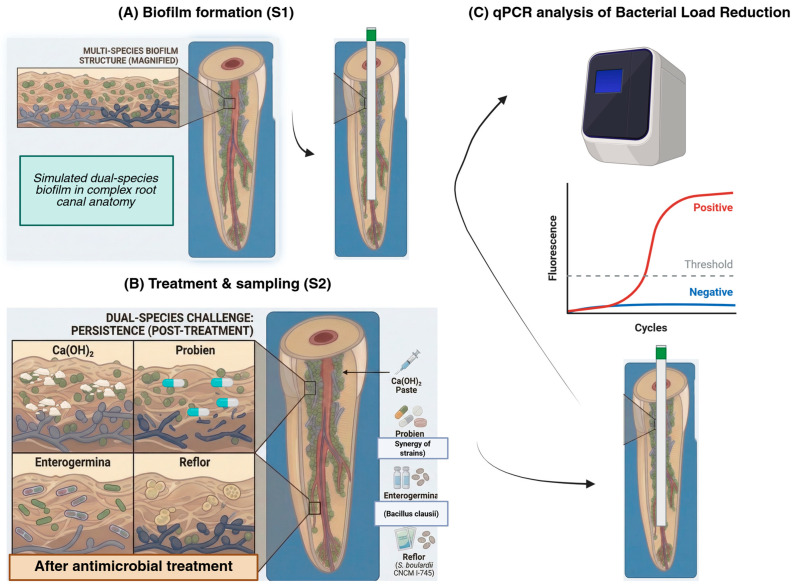
Schematic overview of the experimental workflow.

Prior to the commencement of the study, ethical approval was obtained from the Erciyes University Health Sciences Research Ethics Committee (Approval Date: 22 January 2025; Approval Number: 2025/25). A schematic overview of the experimental workflow, including biofilm formation, probiotic treatment, and qPCR analysis, is presented in Figure 1. A priori power analysis was performed using G*Power software (version 3.1.9.7) to determine the minimum required sample size. Based on the percentage reduction in bacterial load reported in Table 1 of the reference study by Shaaban et al. [20], an effect size of f = 0.60 was estimated. With a significance level (α) of 0.05 and a desired power (1 − β) of 0.95, one-way ANOVA indicated that a minimum of 12 samples per group would be required. While the cited study utilized CFU/mL as a microbiological outcome measure, our study uses qPCR-based quantification of bacterial DNA. Despite methodological differences, the effect size derived from the CFU-based model is deemed appropriate for estimating the expected magnitude of intergroup differences in microbial load. Accordingly, 13 samples were included in each group to meet the power analysis requirements and account for potential variability.

The mesiodistal and buccolingual dimensions of the selected samples were indeed examined radiographically, and round-shaped root canals were confirmed. Teeth that had undergone root canal treatment or showed signs of caries, cracks, or calcification were excluded from the study. Additionally, teeth with multiple canals and root curvature were also excluded. The coronal portion of the teeth was removed using an Isomet 5000 saw (Buehler, Lake Bluff, IL, USA), and the length of the root samples was standardized to 14 mm.

Root canal instrumentation was performed up to size 40.06 with the ProTaper F4 file (Dentsply Maillefer, Ballaigues, Switzerland). The root canals were irrigated with 2 mL of 2.5% NaOCl using a 30-gauge endodontic syringe after each instrument. Following NaOCl irrigation, 10% sodium thiosulfate was applied to neutralize the disinfectant and prevent any lingering effects on subsequent microbiological sampling, in accordance with previously published protocol [22]. Each root’s apical foramen and root surfaces were coated with two layers of nail polish. Each root was placed into a 1.5 mL Eppendorf tube filled with sterile brain heart infusion (BHI) broth (Merck KGaA, Darmstadt, Germany) and autoclaved in the same tube. To verify sterility, the samples were incubated at 37 °C for 48 h and monitored for signs of microbial contamination.

### 2.1. Preparation of Microbial Strains

*E. faecalis*: The ATCC 29212 strain was used. The lyophilized (freeze-dried) stock was incubated at 37 °C under aerobic conditions in BHI medium for 24 h.

*C. albicans*: The ATCC 10231 strain was used. The lyophilized (freeze-dried) stock was incubated in Sabouraud dextrose liquid medium at 37 °C under aerobic conditions for 48 h.

### 2.2. Preparation of Dual-Species Biofilm with E. faecalis and C. albicans

Planktonic cultures of *E. faecalis* grown on blood agar and *C. albicans* grown on Sabouraud dextrose agar (SDA) were used to obtain single colonies, which were inoculated into BHI broth and SDB medium, respectively, and incubated overnight at 37 °C. Following incubation, bacterial and yeast cells were harvested by centrifugation at 4500 rpm for 5 min. The resulting pellets were washed twice with sterile phosphate-buffered saline (PBS; Sigma-Aldrich, St. Louis, MO, USA) and then resuspended in 1× RPMI-1640 (Capricorn Scientific GmbH, Ebsdorfergrund, Germany). The cell density of *E. faecalis* was adjusted spectrophotometrically to an optical density of OD_600_ = 0.10, corresponding to approximately 1.0 × 10^6^ CFU/mL. For *C. albicans*, the cell count was adjusted to 1.0 × 10^6^ CFU/mL using a Neubauer hemocytometer. Cell concentrations were confirmed by plating serial dilutions on appropriate solid media.

For the preparation of mixed-species biofilms of *E. faecalis* and *C. albicans*, cell suspensions of each species were adjusted to a final concentration of 1 × 10^6^ CFU/mL in BHI broth. Equal volumes of the two suspensions were then mixed at a 1:1 ratio and homogenized in sterile tubes [23,24]. CFU calculations were performed solely to standardize the initial microbial inoculum, whereas qPCR-based outcomes were expressed as cell equivalents (CFE) reflecting total microbial DNA; CFU values were not used as outcome measures.

### 2.3. Root Canal Contamination with Dual-Species Biofilm

The tooth specimens were aseptically transferred into sterile Eppendorf tubes. A 10 μL aliquot of the previously prepared 1:1 bacterial and fungal suspension was inoculated into the root canals using a sterile micropipette. To ensure full distribution of the suspension along the entire canal length, a sterile size 15 hand file was used. Each canal was carefully inspected to confirm complete filling. The inoculated roots were then incubated at 37 °C and 95% humidity for four weeks to promote biofilm formation.

During the incubation period, the culture medium was carefully removed every 48 h (every other day) and replaced with fresh sterile growth medium, without additional microbial inoculation, to maintain nutrient availability and sustain the viability of the established biofilm. On the intervening days, culture medium was added to maintain microbial viability, remove dead cells, and prevent nutrient depletion. Dual-species biofilms were initially formed and subsequently allowed to mature in the root canals under controlled incubation conditions. At the end of the incubation period, the specimens were removed from the tubes and rinsed with sterile PBS to eliminate non-adherent cells and residual culture medium. All procedures were carried out under aseptic conditions in a biosafety cabinet using sterile techniques and laminar airflow.

### 2.4. Post-Incubation Period (First Microbial Sampling—S1)

Following a period of four weeks, the specimens were taken out of the inoculation tubes and positioned within biosafety cabinets to prevent contamination, and the initial root canal samples (S1) were obtained. The root canals were irrigated using a 23-gauge needle and sterile saline solution. To sample intracanal contents, three sterile paper points (corresponding to the master apical file size) were inserted to the working length and left in place for 60 s.

This paper point sampling approach was used to collect microbial material from the main canal lumen, including both biofilm-associated microorganisms and planktonic cells released from the biofilm matrix, in accordance with the study objective of quantitatively assessing the overall microbial burden within the root canal system. The paper points were then transferred into sterile Eppendorf tubes containing liquid dental transport medium (LDTM; Oxoid) [25].

### 2.5. Standardization of Probiotic Inoculum

In this study, three commercially available probiotic formulations were used: Probien (capsule form), containing bacterial probiotic strains; Reflor (capsule form), containing the yeast *Saccharomyces boulardii*; and Enterogermina (powder for oral suspension), containing spores of *Bacillus clausii*. To ensure standardization of microbial load across groups, the inoculum was adjusted to approximately 6 × 10^8^ CFU/mL for each formulation. No strain-level isolation or subculturing was performed; instead, microorganisms were directly recovered from the commercial probiotic formulations and processed as whole-product suspensions to reflect their intended commercial composition. For bacterial strains present in Probien and Enterogermina, this standardization was achieved using the 0.5 McFarland standard (≈1.5 × 10^8^ CFU/mL), and combining multiple aliquots of identical concentration solely to obtain the required final volume without altering the CFU/mL. For *S. boulardii* (Reflor), one-quarter of a capsule (≈6 × 10^9^ CFU) was suspended in MRS broth and diluted to the same target concentration. All suspensions were then transferred into sterile centrifuge tubes containing 10 mL of MRS broth and subsequently incorporated into 30% (*w*/*v*) poloxamer 407 gel (300 mg/mL) at a 1:9 ratio, yielding a final poloxamer concentration of approximately 27% (≈270 mg/mL) and a final intracanal preparation of approximately 6 × 10^7^ CFU/mL.

The tubes were vortexed at room temperature for 2 min until a homogeneous suspension was obtained. The suspensions were then incubated at 37 °C for 48 h to promote activation and ensure microbial viability. Following incubation, the preparations were stored at 4 °C in a laboratory refrigerator to preserve cell viability and prevent spontaneous mutation of test strains. All prepared probiotic suspensions were used within 1–2 days.

For intracanal application, the final probiotic mixtures were combined with poloxamer 407 to facilitate delivery into the root canals.

### 2.6. Preparation of Poloxamer Gel

A 30% (*w*/*v*) poloxamer 407 gel was prepared according to a previously described protocol [20].

### 2.7. Experimental Groups

- Group 1: Probien (n = 13)
- Group 2: Enterogermina (n = 13)
- Group 3: Reflor (n = 13)
- Group 4: Calcium Hydroxide (n = 13)

For Groups 1 to 3, 1 mL of the corresponding probiotic suspension was mixed with 9 mL of 30% poloxamer 407 gel (prepared in MRS broth) to obtain a homogeneous mixture. The prepared probiotic–poloxamer gel formulations were then delivered into root canals that had been previously contaminated with mature dual-species (*E. faecalis* and *C. albicans*) biofilms.

In Group 4, UltraCal XS calcium hydroxide paste (Ultradent Products, South Jordan, UT, USA) was placed in the root canals at working length using a 29-gauge (0.33 mm) NaviTip and delivered into the canal until it reached the level of the canal orifice.

After intracanal placement of the materials in all experimental groups, a sterile teflon pellet was placed, and the coronal surface of the roots was then sealed with at least 2 mm of temporary filling material (Ultratemp, Ultradent). All specimens were then incubated at 37 °C for 1 week to allow interaction between the medicaments and the intracanal biofilms.

### 2.8. Control Groups

- Group 5: Positive Control (Infected Control Group) (n = 13)

Root canals were contaminated with mature dual-species biofilms, but no intracanal medicament was applied.

- Group 6: Negative Control (Sterile Control Group) (n = 13)

Root canals were not contaminated with any biofilm and received no intracanal medicament. In both control groups, the root canals were similarly sealed coronally and incubated at 37 °C for 1 week under respective conditions. Between S1 and S2 sampling points, no mechanical instrumentation or additional chemomechanical procedures were performed. Following S1 sampling, intracanal medicaments were applied directly into the canals and left undisturbed for a 7-day period. This design was intentionally chosen to isolate and evaluate the antimicrobial effect of the intracanal medicaments alone, independent of the confounding influence of mechanical preparation or irrigant activation.

The tooth samples were randomly allocated into experimental and control groups using a computer-generated random sequence. No sample loss occurred during the study. Root canal preparation procedures were performed by a single operator (B.Y.), while microbiological laboratory analyses and raw data collection were independently conducted by other investigators (S.T.Ü. and N.Y.), who were blinded to the group allocation.

### 2.9. Post-Intervention Period (Second Sampling—S2)

After the 1-week intervention period, a second microbiological sampling (S2) was performed to evaluate the bacterial load after probiotic application. At the end of this period, intracanal medicaments were removed using a standardized irrigation protocol applied to all experimental groups. Each root canal was irrigated with 6 mL of 17% ethylenediaminetetraacetic acid (EDTA), followed by 6 mL of sterile saline, in accordance with previously published protocol [26]. In the calcium hydroxide group, an additional irrigation step with 1 mL of 0.5% citric acid solution was performed to neutralize residual calcium hydroxide, followed by a final rinse with 3 mL of sterile saline, as described in a previous study [27]. This additional step was included to prevent potential interference of residual medicament with subsequent qPCR-based microbial analysis. Meticulous care was taken to ensure complete removal of the medicament in order to eliminate any potential interference with subsequent microbiological analysis. After ensuring the complete removal of the medicaments, microbiological sampling was performed by inserting three sterile paper points into each canal for 60 s to collect the S2 samples. All samples were processed immediately or stored at −20 °C prior to DNA extraction to preserve DNA integrity.

### 2.10. DNA Extraction and Quantification

Genomic DNA was extracted from root canal samples collected at two time points (S1 and S2) using a commercial genomic DNA mini kit PureLink™ (İnvitrogen, Carlsbad, CA, USA, Catalog No. K1820-00) following the manufacturer’s instructions. Although dual-species biofilms were formed using a 1:1 mixture of *E. faecalis* and *C. albicans*, DNA concentration and purity were assessed by measuring absorbance at 260 nm using a NanoDrop ND-1000 spectrophotometer (Thermo Fisher Scientific, Waltham, MA, USA).

### 2.11. Quantitative Real-Time PCR (qPCR) Protocol

The antibacterial effects of different commercial probiotic formulations on a dual biofilm model consisting of *E. faecalis* and *C. albicans* strains were determined by assessing changes in bacterial loads using quantitative real-time PCR (qPCR).

Target-specific primers were used for the detection of *E. faecalis* and *C. albicans* in the qPCR reactions applied to S1 and S2 samples obtained from the root canal dual biofilm model. For *E. faecalis*, primers targeting the 16S region were used: forward 5′-AGAGTTTGATCCTGGCTCA-3′ and reverse 5′-GGTTACCTTGTTACGACTTC-3′, as described by Mahfouz Omer et al. [25]. For *C. albicans*, the primer pair described by Nouroloyouni et al. [28] was used: forward 5′-TTTCTCTCGCCCCGTGTGGGT-3′ and reverse 5′-GGCAGCTCTACCTTCAACGCCA-3′.

The SYBR Green-based detection was conducted to quantify bacteria in biofilm samples. Each reaction was performed in a volume of 20 μL; 10 μL of 1X SYBR Green Mix (Jena Biosciences, Jena, Germany), 300 nM concentration of each forward and reverse primer, and 2 μL of template DNA were added, followed by sterile nuclease-free water up to the reaction volume. DNA of standard bacterial isolates *E. faecalis* (ATCC 29212) and *C. albicans* (ATCC 10231) were extracted and included in the reaction as positive controls. No-template controls (NTCs), in which sterile nuclease-free water was substituted for DNA, were included as negative controls. qRT-PCRs were performed in real time on a Rotor Gene Q 5Plex HRM Real Time PCR (Qiagen, Hilden, Germany). The reaction mixture was first incubated at 95 °C for 5 min. Amplification was carried out for 45 cycles of denaturation at 95 °C for 20 s and annealing at 55 °C for 1 min. Melting curve analysis was performed after amplification. Concerning the melting curve, a thermal gradient was applied from 55–60 °C to 95 °C for *E. faecalis* and *C. albicans* at 0.5 °C/5 s. The efficacy of each reaction was determined using this formula: E = (10^(1/slope)^ − 1). All the reactions were performed twice.

Bacterial cell equivalent counts were extracted for each sample based on a standard curve generated from a 10-fold dilution (from 10^0^ to 10^8^ cells) of DNA extracted from *E. faecalis* (ATCC 29212) and *C. albicans* (ATCC 10231).

### 2.12. CFU-Based Standardization for qPCR-Derived CFE Quantification

For each of the *E. faecalis* and *C. albicans*, liquid cultures were washed in PBS and standardized to their absorbance at 600 nm reaching a value of 1 (1 × 10^8^ CFU/mL), prior to serially diluting in preparation for calibrated standard curve analysis. These cultures were then diluted 10-fold 8 times. Subsequently, 10 μL from each of the dilutions was cultured on an agar medium. The number of CFUs was counted on plates with dilutions that yielded between 30 and 300 CFUs.

### 2.13. Construction of a Standard Curve

Total bacterial genomic DNA was extracted from each dilution. Quantities of the genomic DNA (variable a) were equated to bacterial CFUs (variable b) as follows: Using an example, b = a × (*E. faecalis* CFU in dilutions). A standard curve was constructed using a scatter plot where the x-axis represented bacterial CFUs and the y-axis represented the corresponding Ct values. The linearity of the standard curve was assessed by calculating the coefficient of determination (R^2^) using GraphPad Prism V5 (GraphPad Software Inc., San Diego, CA, USA).

The bacterial counts were determined using standard graphics according to cycle threshold values. Baseline threshold values of the samples were adjusted to correspond with the equivalent standard curve; Ct values were then used to approximate the number of corresponding colony-forming equivalents (CFEs) based on standard curves created from serial 10-fold dilutions of each bacterial species. Standard curves were prepared for *E. faecalis* and *C. albicans*; R^2^ values ranged from 0.92 to 0.99. qPCR efficiency is found as 1.17 for *E. faecalis* and 0.44 for *C. albicans*.

Changes in bacterial load (reduction or increase) in response to different experimental procedures (S1–S2) were calculated using the following formula:

Percentage of Bacterial Fold Change = (Initial FC − Post-treatment FC)/Initial FC × 100

(where FC refers to fold change in bacterial gene expression).

### 2.14. Statistical Analysis

All statistical analyses were conducted using two complementary approaches, depending on the data structure. The distribution of variables was first examined with the Shapiro–Wilk test. For CFE values (expressed as mean ± SE and transformed to the 10^n^ scale), comparisons across groups, microorganisms, and time points were performed using robust analysis of variance (Robust ANOVA with 5% trimmed means, WRS2 package in R, version 4.4.0). Post hoc pairwise comparisons were carried out using Holm-adjusted robust *t*-tests, and results were presented as trimmed mean ± standard error.

For ΔCt values and percentage reduction data (expressed as median and range), non-parametric tests were applied. Intergroup comparisons were performed using the Kruskal–Wallis test, followed by Dunn’s post hoc test for multiple comparisons. These analyses were conducted using IBM SPSS Statistics (version 23). A *p*-value < 0.05 was considered statistically significant.

## 3. Results

A total of six groups (*n* = 13 per group; overall n = 78) were analyzed, and qPCR-based quantitative assessment was performed for *E. faecalis* and *C. albicans* at two time points (S1: post-incubation; S2: post-treatment). As expected, no amplification was detected in the negative control group at either time interval, confirming sterility throughout the study.

Positive ΔCt values indicate a decrease in microbial load, whereas negative ΔCt values represent an increase (Table 1). For *E. faecalis*, no statistically significant intergroup differences were observed in ΔCt distributions (Kruskal–Wallis, *p* = 0.17). In contrast, ΔCt analysis for *C. albicans* revealed significant intergroup variation (*p* < 0.001), with the calcium hydroxide group showing the most pronounced reduction compared to other groups (Table 1). Effect size analysis revealed that intergroup differences for *C. albicans* ΔCt were large (H = 24.55, *p* < 0.001), while *E. faecalis* ΔCt showed negligible effect sizes (H = 6.42, *p* = 0.170). These findings were further supported by analyses of absolute CFE values at S1 and S2.

CFE values (CFE/mL) were reported as mean ± SE at both S1 and S2 time points. For *E. faecalis*, intergroup differences were not statistically significant at either S1 (*p* = 0.12) or S2 (*p* = 0.21). For *C. albicans*, a significant difference was observed among groups at S1 (*p* = 0.018), whereas no significant difference was detected at S2 (*p* = 0.145) (Table 2). It should be noted that CFE distributions were markedly right-skewed and contained outliers; therefore, timepoint-based group means (Table 2) and within-sample median changes may provide different impressions. Importantly, mean CFE values presented in Table 2 reflect group-level averages at each time point, whereas percentage reduction values in Table 3 are derived from within-sample changes. As a result, these metrics may yield different apparent trends, particularly in the presence of skewed data and outliers. The wide range observed in percentage reduction values is primarily attributable to the mathematical sensitivity of relative change calculations to baseline differences, particularly in low-abundance samples. In some samples, extremely high percentage change values were observed, particularly in cases with very low baseline (S1) CFE values. These extreme values are attributable to the mathematical sensitivity of ratio-based calculations when baseline values approach zero and should be interpreted as computational artifacts rather than true biological effects. Therefore, mean-based metrics may exaggerate variability, whereas median ΔCt values and non-parametric analyses provide a more robust and biologically meaningful representation of antimicrobial effects. For biological interpretation, measures less influenced by extreme values—such as median ΔCt or microbial reduction percentage—are considered more reliable.

Percentage reduction was calculated using the formula (S2–S1)/S1 × 100 (Table 3). For *E. faecalis*, no significant intergroup differences were observed (*p* = 0.17). In contrast, analysis for *C. albicans* revealed significant differences among groups (*p* = 0.001), with the calcium hydroxide group showing the most pronounced reduction compared to the others (Table 3). Effect size estimates indicated a strong effect for *C. albicans* % reduction (H = 19.85, *p* < 0.001), but negligible effect sizes for *E. faecalis* % reduction (H = 6.36, *p* = 0.170).

## 4. Discussion

Endodontic infections are increasingly recognized as inflammatory dysbiotic conditions in which pathogenic microorganisms dominate biofilm communities [29]. In contrast, healthy endodontic microbiota are characterized by low-virulence commensal species that help maintain host–microbial balance. Accordingly, the deliberate introduction of beneficial microorganisms to restore this balance represents a promising biological approach to endodontic disinfection. In this context, the present study evaluated the inhibitory effects of probiotic formulations against a dual-species biofilm model composed of *E. faecalis* and *C. albicans*, two microorganisms frequently associated with persistent root canal infections.

Persistent endodontic infections are strongly associated with resistant microorganisms, particularly *E. faecalis* and *C. albicans*, which are frequently detected in refractory cases [30,31]. Dual-species biofilms formed by these organisms are known to exhibit increased structural complexity and resistance to antimicrobial agents compared with single-species biofilms [32,33]. Therefore, a dual-species biofilm model was employed in this study to simulate persistent infections and evaluate treatment strategies against resistant pathogens.

Previous studies have shown that *E. faecalis* biofilms matured for 21 days in dentin canals exhibit significantly greater resistance to irrigants than younger biofilms [34], and mixed-species biofilms containing *E. faecalis*, *C. albicans*, and *S. gordonii* have been successfully established over a 21-day period to model persistent infections [23]. In the present study, a four-week-old dual-species biofilm model (*E. faecalis* and *C. albicans* at equal ratios) was employed to ensure maturity and to closely mimic the structural complexity and resistance of persistent endodontic infections. Although a four-week incubation period was employed to promote biofilm maturity based on established protocols, no direct morphological or structural verification (e.g., SEM or CLSM) was performed. Therefore, biofilm maturity was inferred from incubation time, controlled growth conditions, and prior literature rather than direct imaging. Future studies incorporating imaging-based validation methods would provide additional confirmation of biofilm architecture and inter-sample comparability. All medicaments were maintained in the canals for one week, as this duration is required for calcium hydroxide to exert its antimicrobial effect [35] and has also been used in a probiotic study, where probiotics demonstrated effectiveness within this period [20]. This approach allowed consistent comparison across groups while minimizing methodological bias from differing application times.

Intracanal probiotic formulations were prepared following the framework of Ravi et al. [19], using commercially available products incorporated into poloxamer 407 gel. Probiotics were evaluated as intact formulations to preserve translational relevance, and the inoculum was standardized by bacterial count (≈6 × 10^8^ CFU/mL) rather than weight to account for variability in excipients. The final intracanal preparation (≈6 × 10^7^ CFU/mL; 270–300 mg/mL poloxamer) was designed to enable comparable microbial loading and sustained release within the root canal system [36].

In the present study, neither calcium hydroxide nor the tested commercial probiotic preparations achieved statistically significant inhibition against *E. faecalis* (*p* = 0.17). This finding is consistent with previous reports highlighting the adaptive resistance of this pathogen to commonly used intracanal medicaments. For example, calcium hydroxide has been shown to exhibit limited effectiveness against *E. faecalis*, largely due to its ability to tolerate alkaline environments and persist within the root canal system [16,37]. Additionally, the antimicrobial efficacy of calcium hydroxide may vary depending on the carrier vehicle used [38]. Collectively, these findings underscore the limitations of calcium hydroxide and suggest that it may be insufficient when used alone against this highly resilient pathogen.

A similar lack of significant inhibition was observed with the tested probiotic preparations, but the reasons appear to be fundamentally different. It is important to note that the probiotic formulations evaluated in the present study differ substantially in their microbial composition, which may influence their mechanisms of antimicrobial action. Probien contains *Lactobacillus* and *Bifidobacterium* species, which are known to exert antimicrobial effects primarily through the production of organic acids, bacteriocins, and competitive exclusion of pathogens. In contrast, Enterogermina contains *Bacillus clausii* spores, which may act through the production of antimicrobial peptides and modulation of the local microenvironment. Reflor contains the yeast *Saccharomyces boulardii*, which has been reported to interfere with pathogen adhesion and biofilm formation, as well as to modulate microbial signaling pathways. These mechanistic differences have been described in previous studies on probiotics in oral and systemic contexts [39]. However, under the conditions of the present study, no significant differences in antimicrobial efficacy were observed among the tested formulations. This suggests that, despite their distinct biological properties, these commercially available probiotic products may exhibit similarly limited antimicrobial effects within the specific ecological conditions of the root canal system.

Previous studies have reported antimicrobial effects of probiotics against *E. faecalis*; however, important methodological differences may explain the discrepancy with our findings. For example, Ravi et al. [19] used disk diffusion assays, which primarily reflect planktonic susceptibility under simplified conditions, whereas the present study evaluated mature dual-species biofilms using a qPCR-based approach, providing a more stringent and clinically relevant assessment. Similarly, Shaaban et al. [20] employed cell-free supernatants (CFS) containing pre-formed antimicrobial metabolites, whereas our study used viable probiotic formulations that require survival, colonization, and in situ metabolite production within the root canal environment. Taken together, these differences in experimental models and probiotic formulations likely account for the divergent outcomes.

In the present study, calcium hydroxide demonstrated significantly greater antifungal activity against *C. albicans* compared with both probiotic groups and the positive control (*p* < 0.001), consistent with previous reports. This effect may be related to the use of a propylene glycol–based formulation, which enhances dentinal penetration and antimicrobial efficacy [40]. However, conflicting evidence also exists, as *C. albicans* has been shown to tolerate high pH and resist calcium hydroxide under certain conditions [41]. These findings suggest that the antifungal effectiveness of calcium hydroxide is influenced by multiple factors, including the carrier, exposure time, and experimental model.

Although previous studies have suggested antifungal effects of probiotics against *C. albicans*, methodological differences limit comparability with our findings. For example, Bohora & Kokate [21] evaluated culture-derived supernatants without including viable probiotic cells, while Widyarman and Lazaroni [42] used early-stage biofilm models. In contrast, the present study assessed viable, commercially available probiotics against mature dual-species biofilms within standardized root canals. These differences in experimental design and probiotic formulations likely explain the absence of significant antifungal effects compared to calcium hydroxide in our study.

A qPCR-based approach was employed to enable sensitive quantification of total microbial load, including non-culturable microorganisms [43]. Compared with Fonseca et al. [44], who used polymicrobial DNA and a single standard curve, our study applied a defined dual-species biofilm model with separate standard curves for each organism, allowing more accurate species-specific quantification.

The sampling design was structured to isolate the effects of intracanal medicaments by eliminating the influence of chemomechanical preparation, with S2 measurements performed after a 7-day incubation period. In the positive control group, root canals were sealed and incubated for 7 days without nutrient supplementation, which may have induced ecological shifts such as microbial adaptation or changes in species dominance. However, these conditions were applied uniformly across all groups, ensuring that any such changes occurred consistently. Therefore, intergroup differences can be attributed primarily to the effects of intracanal medicaments rather than environmental variability. This setup also reflects the closed, nutrient-limited nature of the root canal system in vivo. It should be noted that qPCR quantifies total microbial DNA rather than viable cells; however, it was selected as the primary outcome measure due to its sensitivity and its ability to capture overall microbial burden in mature biofilms.

This study has certain limitations. As with all in vitro biofilm models, the experimental setup cannot fully replicate clinical conditions. Endodontic infections are inherently polymicrobial [45], whereas the present model was restricted to two species. Although *E. faecalis* and *C. albicans* are among the most frequently detected and resistant microorganisms, a dual-species model does not fully capture the complexity of polymicrobial biofilms, including interspecies interactions that may influence antimicrobial response. Therefore, the findings should be interpreted as reflecting a simplified but clinically relevant model rather than the full spectrum of endodontic infections. Future studies using multispecies models are warranted.

Second, although qPCR provides sensitive and quantitative data, it does not differentiate between viable and non-viable bacteria [46], which may lead to an overestimation of microbial persistence. Therefore, the observed reductions should be interpreted as changes in total microbial DNA load rather than direct evidence of viable cell elimination. Baseline microbial load (S1) was standardized; however, minor variability among specimens cannot be entirely excluded due to anatomical differences. While no significant intergroup differences were observed at baseline for *E. faecalis*, slight variability was detected for *C. albicans*. To account for this, ΔCt (S2–S1) values were used, allowing each specimen to serve as its own control and reducing the impact of baseline variability. Despite these limitations, qPCR was selected for its high sensitivity and ability to detect both culturable and non-culturable microorganisms within mature biofilms. Future studies incorporating viability-based approaches, such as PMA-qPCR, may provide further insight into viable microbial dynamics. Additionally, paper point sampling may underestimate microbial load in complex canal anatomies [47].

It is evident from this study that intracanal biofilms, especially those formed by resistant species like *E. faecalis*, cannot be eradicated by medicaments alone, highlighting the necessity of combined chemomechanical strategies. Similar observations were reported by Zancan et al. [48], who demonstrated that calcium hydroxide is not effective against microbial biofilms. For pathogens such as *E. faecalis*, which have been shown in previous studies to adapt to the harsh conditions of the root canal and exhibit resistance to antimicrobial medicaments, greater emphasis should be placed on physically disrupting biofilms through chemomechanical preparation. Relying solely on medicaments for disinfection is therefore not a viable treatment strategy. Instead, effective canal disinfection can only be achieved through a balanced integration of both chemical and mechanical approaches.

In a previous study by our research team, *Bifidobacterium longum* demonstrated natural colonization of root canals preconditioned with calcium hydroxide [49], suggesting that probiotic establishment may depend on the ecological conditions created by prior intracanal treatment. In line with this, the present findings indicate that commercially available probiotic formulations are unlikely to be effective as standalone intracanal medicaments. However, based on the current results and limited previous evidence, probiotics may have potential as adjunctive agents following chemomechanical preparation, where reduced microbial load and altered ecological conditions may favor colonization. Further studies are needed to determine the optimal timing of probiotic application and the role of factors such as pH modulation in intracanal colonization.

These findings suggest that probiotic formulations originally developed for gastrointestinal use may not be directly suitable for the unique ecological conditions of the root canal system. However, this limitation should be interpreted in the context of the specific formulations tested in the present study rather than as a general constraint of probiotic-based approaches in endodontics. Future research should focus on the development of oral-specific probiotic formulations and optimized delivery systems, such as encapsulation or tailored carriers, to ensure stability, targeted delivery, and sustained activity within the root canal environment. Such approaches may facilitate the development of biologically oriented adjunctive strategies to improve outcomes in persistent endodontic infections.

## Figures and Tables

**Table 1:** Comparison of ΔCt (S2–S1) values among treatment groups.

Probien	Reflor	Enterogermina	Ca(OH)_2_	Positive Ctrl	*p*
*E. faecalis* ΔCt (S2–S1)					
−0.86 (−3.01–7.5)	−0.3 (−3.17–3.83)	−0.85 (−8.86–2.72)	0.15 (−2.63–2.76)	0.6 (−1.98–4.33)	0.17
*C. albicans* ΔCt (S2–S1)					
3.07 (1.42–5.03) ^bb^	4.5 (2.09–6.02) ^ab^	4.85 (0.5–7.51) ^ab^	8.19 (−1.75–12.19) ^a^	3.33 (1.28–4.69) ^b^	<0.001 *

Note: Values are presented as median (minimum–maximum). ΔCt = (Ct at S2) − (Ct at S1). Statistical comparisons were performed using the Kruskal–Wallis test. Groups sharing the same superscript letter do not differ significantly. * Statistically significant difference (*p* < 0.05). Positive ΔCt values indicate a decrease in microbial load, whereas negative values indicate an increase.

**Table 2:** Comparison of CFE (CFE/mL) values (mean ± standard error) among treatment groups at S1 and S2 sampling times.

Time	Probien	Reflor	Enterogermina	Ca(OH)_2_	Positive Ctrl	*p*-Value
*E. faecalis* (mean ± standard error)						
S1	(3.59 ± 2.73) × 10^6^	(3.61 ± 1.65) × 10^6^	(2.51 ± 1.00) × 10^6^	(9.95 ± 2.55) × 10^5^	(2.05 ± 1.23) × 10^6^	0.12
S2	(2.03 ± 1.85) × 10^7^	(3.12 ± 0.72) × 10^6^	(1.07 ± 0.68) × 10^7^	(4.17 ± 3.29) × 10^6^	(4.02 ± 1.15) × 10^5^	0.21
*C. albicans* (mean ± standard error)						
S1	(6.07 ± 0.51) × 10^5^	(6.34 ± 0.61) × 10^5^	(5.38 ± 0.75) × 10^5^	(4.73 ± 1.02) × 10^5^	(4.66 ± 0.84) × 10^5^	0.018 *
S2	(2.12 ± 0.28) × 10^5^	(1.37 ± 0.19) × 10^5^	(1.16 ± 0.33) × 10^5^	(3.49 ± 1.17) × 10^4^	(1.58 ± 0.46) × 10^5^	0.145

Note: CFE = Colony-Forming Equivalents. * Indicates statistically significant differences (*p* < 0.05) among groups.

**Table 3:** Microbial reduction percentage in *E. faecalis* and *C. albicans* across treatment groups.

Probien	Reflor	Enterogermina	Ca(OH)_2_	Positive Ctrl	*p*-Value
*E. faecalis* reduction %					
19.93% (−99.99–3632%)	43.43% (−98.99–4422.3%)	210.45% (−96.2–4,233,924.7%)	−10.66% (−96.38–2262.5%)	−51.4% (−99.45–981.3%)	0.17
*C. albicans* reduction %					
−67.63% (−84.25–40.64%) ^b^	−80.35% (−89.04–53.6%) ^ab^	−83.16% (−93.66–16.78%) ^ab^	−94.44% (−98.86–90.18%) ^a^	−70.56% (−82.14–37.51%) ^b^	0.001 *

Note: Reduction (%) was calculated as (S2 − S1)/S1 × 100. * and Values with different superscript letters indicate statistically significant differences among groups (*p* < 0.05), while values sharing the same letter are not significantly different.

## Data Availability

The authors declare that the raw data of this study are available from the corresponding author upon reasonable request.
